# Peripheral CD4 memory T cells predict the efficacy of immune checkpoint inhibitor therapy in patients with non-small cell lung cancer

**DOI:** 10.1038/s41598-023-37736-3

**Published:** 2023-07-04

**Authors:** Minehiko Inomata, Masahiro Matsumoto, Naoki Takata, Kana Hayashi, Zenta Seto, Takahiro Hirai, Kotaro Tokui, Chihiro Taka, Seisuke Okazawa, Kenta Kambara, Shingo Imanishi, Toshiro Miwa, Ryuji Hayashi, Shoko Matsui, Kazuyuki Tobe

**Affiliations:** 1 First Department of Internal Medicine, Toyama University Hospital, Toyama, Japan; 2 Department of Clinical Oncology, Toyama University Hospital, Toyama, Japan

**Keywords:** Cancer, Lymphocytes

## Abstract

Immune checkpoint inhibitors have significantly improved the prognosis in patients with non-small cell lung cancer, compared with cytotoxic agents. However, the prediction of treatment response is often difficult, even after assessing the tumor programmed death-ligand 1 expression. We conducted this observational study to analyze the association between the differentiation of peripheral CD4 + T cells and the efficacy of immune checkpoint inhibitor therapy. We enrolled patients who were diagnosed with non-small cell lung cancer and received immune checkpoint inhibitor therapy between 2020 and 2022. Blood samples were collected at the start of immune checkpoint inhibitor therapy, and the expressions of PD-1, CCR7, and CD45RA in peripheral CD4 + T cells were analyzed by flow cytometry. The association between the findings of flow cytometry and survival after the initiation of the immune checkpoint inhibitor therapy was evaluated. Forty patients with non-small cell lung cancer were enrolled. The Cox proportional hazards model showed that an increased proportion of CD45RA-CD4 + T cells was associated with a reduced risk of progression after adjustment for performance status, tumor programmed death-ligand 1 expression level, mutation status of the epidermal growth factor receptor gene, and combined therapy with cytotoxic agents. The present study showed that the proportion of peripheral CD45RA- CD4 + T cells was associated with progression-free survival after the initiation of immune checkpoint inhibitor therapy, independent of several clinical factors.

## Introduction

Systemic therapy with a cytotoxic agent has been the standard treatment for advanced non-small cell lung cancer (NSCLC) with negative driver mutations. In a phase III trial comparing the effectiveness of cisplatin plus pemetrexed versus cisplatin plus gemcitabine in NSCLC, the 2-year survival rates were 18.9% and 14.0%, respectively^1^. However, immune checkpoint inhibitors (ICI) have conferred longer survival. Combination therapy with pembrolizumab and cytotoxic agents showed a 2-year survival rate of 45.7% in NSCLC^2^. Furthermore, pembrolizumab monotherapy demonstrated a 5-year survival rate of 31.9% in NSCLC patients with strong tumor programmed death-ligand 1 (PD-L1) expression^3^.

Tumor PD-L1 expression is related to the therapeutic efficacy of ICIs, with higher survival rates observed in patients with strong PD-L1 expression. However, early progression is observed even in patients with positive PD-L1 expression^4^, while some patients with negative PD-L1 expression show long-term survival^5^. Tumor PD-L1 expression is reported to be heterogeneous in tumor tissue, and it might be modified by treatment exposure, in addition to being caused by multiple mechanisms^6^. Moreover, it is problematic that tissue samples are required for the evaluation of PD-L1 expression. Therefore, investigation for biomarkers that complement tumor PD-L1 expression is underway. Peripheral immune cells are one of them, including lymphocytes^7–11^, monocytes^12^, and eosinophils^13^.

We previously reported that the proportion of peripheral PD-1 + CD4 + T cells evaluated within 1 week after the initiation of ICIs is associated with progression-free survival (PFS)^14^. Furthermore, previous studies have reported that peripheral PD-1 + CD4 + T lymphocytes might correspond to effector memory T cells^15,16^. Thus, we believe that T-cell differentiation, classified as naive T cells, effector memory T cells, central memory T cells, and effector T cells, is associated with the efficacy of ICI therapy. For example, Zuazo M et al. reported that differentiated CD4 + T cells in peripheral blood, defined as CD27-CD28-CD4 + T cells, were associated with longer PFS after the initiation of ICIs in patients with NSCLC.

We conducted this observational study to analyze the association between the differentiation of CD4 + T cells, including naive, effector memory, central memory, and effector T cells, in the peripheral blood of patients with NSCLC and the efficacy of ICI therapy.

## Methods

### Patients

Patients diagnosed with NSCLC and receiving ICI therapy at Toyama University Hospital between 2020 and 2022 were prospectively enrolled in this study. Selection criteria were established as follows: (1) Patients who were cytologically or histologically diagnosed with NSCLC, including adenocarcinoma or squamous cell lung cancer; (2) Patients who were eligible for systemic therapy and would receive ICI therapy, including ICI monotherapy or combination therapy with cytotoxic anticancer agents. Patients who had been treated with ICI therapy before the study period were also eligible. However, patients diagnosed with neuroendocrine cancer, sarcomatoid carcinoma, or poorly differentiated NSCLC were excluded.

### Clinical examinations and treatments

The evaluation of driver gene mutation and the tumor PD-L1 expression was commissioned to commercial laboratories, and the information were retrieved from medical charts. The absence or existence of driver gene mutation was evaluated by PCR, immunohistostaining, or next-generation sequencing, and the PD-L1 tumor proportion score (TPS) was assessed using the 22C3 antibody. Clinical information, including age, performance status (PS), disease stage, and the neutrophil–lymphocyte ratio (NLR) at the initiation of the treatment with ICIs, were assessed. The disease stage was re-staged based on the most recent imaging examination before the treatment. Therapeutic regimens, withdrawal, and discontinuation of the treatment were determined by clinical judgment. Disease progression was defined as either tumor growth or newly emerging lesions according to the Response Evaluation Criteria outlined in Solid Tumors version 1.1 or based on clinical judgment of exacerbation.

### Flow cytometry

Peripheral blood was collected at the initiation of ICI therapy. The antibodies used in this study were anti-CD3-Fluorescein isothiocyanate (clone HIT3a), anti-CD4-allophycocyanin/Cy7 (clone A161A1), anti-PD1 (CD279)-phycoerythrin/Cy7 (clone EH12.2H7), anti-CCR7-PE (clone G043H7), and anti-CD45RA-APC (clone HI100) (BioLegend, San Diego, CA, USA). Flow cytometry was contracted to SRL Inc (Tokyo, Japan), performed using whole blood.

### Statistical analysis

The endpoints of this study were PFS and overall survival (OS), after the initiation of ICI therapy. PFS events were defined as progression or death and were censored at the date of the last visit or the start of the next treatment without any events. The OS event was death and was censored at the date of the last visit without death. Survival analysis was performed after a 3-month observation period from the last patient enrollment.

Patients were subdivided according to the median of the proportion of each subset against CD3 + CD4 + T cells, including CCR7 + CD45RA + CD4 + T cells (naive T cells), CD45RA-CD4 + T cells (memory T cells), CCR7 + CD45RA-CD4 + T cells (central memory T cells), CCR7-CD45RA-CD4 + T cells (effector memory T cells), and CCR7-CD45RA + CD4 + T cells (effector T cells). The cutoff value of the NLR was set at 5, based on previous reports^17^.

Survival among the subdivided patient groups was analyzed by univariate analysis using the log-rank test and multivariate analysis using the Cox proportional hazard model, adjusting for PS, PD-L1 expression level, epidermal growth factor receptor (EGFR) mutation status, and combined therapy with cytotoxic agents. Fisher's exact test was used to test the nominal scale. A p-value of less than 0.05 was considered significant. Statistical analysis was performed using JMP version 15.0.0 (SAS, Cary, USA).

### Ethical approval

This study was conducted in compliance with the Declaration of Helsinki and the Ethical Guidelines for Medical and Biological Research Involving Human Subjects (Ministry of Health, Labour and Welfare, Japan), with the approval of the Ethics Committee, University of Toyama (approval number: R2020042).

### Consent to participate

Informed consent was obtained from all individual participants included in the study.

## Results

### Patient characteristics

A total of the 40 patients were enrolled. PD-L1 TPS was confirmed as < 1%, 1–49%, and ≥ 50% in 11 (27.5%), 18 (45.0%), and 10 (25.0%) patients, respectively. The EGFR mutation was detected in 6 patients, and 3 of them were treated with EGFR-TKIs before the treatment with ICIs. The KRAS mutation was detected in 5 patients, who were treated with ICIs as a first-line treatment (Table 1).

**Table 1:** Patient characteristics.

	N	%
Whole	40	
Age (yr)		
< 75	18	45.0
≥ 75	22	55.0
Sex		
Male	31	77.5
Female	9	22.5
Smoking history		
Yes	34	85.0
No	6	15.0
Histology		
Adenocarcinoma	28	70.0
Squamous cell carcinoma	12	30.0
Driver mutation		
EGFR	6	15.0
KRAS	5	12.5
Negative/unknown	29	72.5
PD-L1 TPS		
< 1%	11	27.5
1–49%	18	45.0
≥ 50%	10	25.0
Unknown	1	2.5
PS		
0–1	33	82.5
2	7	17.5
Stage		
2B	1	2.5
3B	2	5.0
4A	23	57.5
4B	14	35.0
Treatment		
PD-1 inhibitors	20	50.0
PD-L1 inhibitors	1	2.5
PD-1 inhibitors + CTLA-4 inhibitors	11	27.5
Cytotoxic agents + PD-1 inhibitors	3	7.5
Cytotoxic agents + PD-L1 inhibitors	2	5.0
Cytotoxic agents + PD-1 inhibitors + CTLA-4 inhibitors	2	5.0
Cytotoxic agents + VEGF inhibitors + PD-L1 inhibitors	1	2.5
Treatment line of ICI		
1	34	85
≥ 2	6	15

EGFR, epidermal growth factor receptor; ICI, immune checkpoint inhibitor; PD-L1 TPS, programmed death ligand-1 tumor proportion score; PS, performance status; VEGF, vascular endothelial growth factor.

### Survival

**Figure 1:**
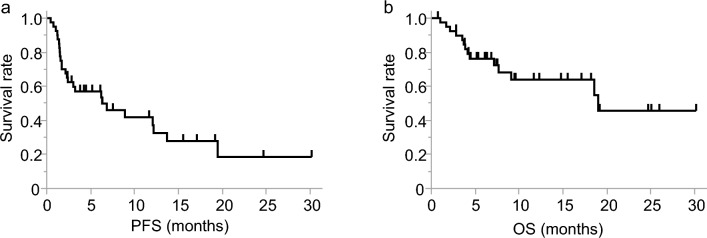
Progression-free survival (**a**) and overall survival (**b**) after the initiation of immune checkpoint inhibitor therapy in overall population.

The median (95% confidence interval, CI) of PFS after the initiation of ICI therapy in 40 patients was 6.3 months (2.3–12.2 months), and the median (95% CI) of OS was 19.0 months (7.7–not estimated). PFS and OS events were observed in 25 and 14 patients, respectively (Fig. 1).

**Figure 2:**
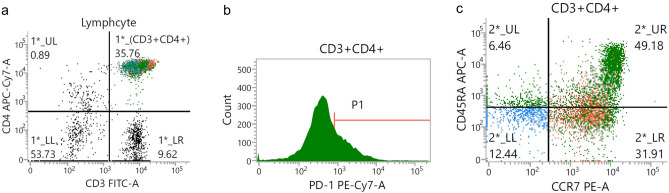
Representative flow cytometry plots of T cells according to CD3-CD4 (**a**), PD-1 (**b**), and CD45RA-CCR7 (**c**) expression profiles. PD-1, programmed death-1.

**Figure 3:**
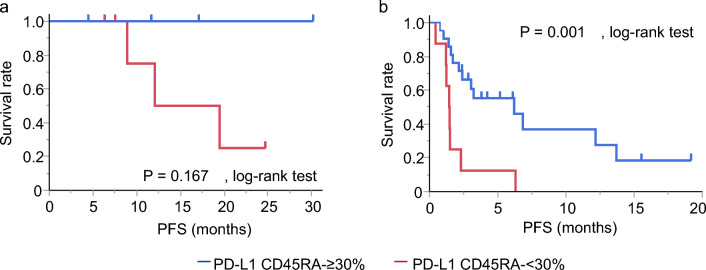
Progression-free survival in patients with a higher (≥ 30%) and lower (< 30%) proportion of CD45RA-CD4 + T cells in each group classified according to tumor PD-L1 expression level of ≥ 50% (**a**) and < 50% (**b**). PD-L1, programmed death ligand-1.

Representative flow cytometry plots of T cells according to CD3-CD4, PD-1, and CD45RA-CCR7 expression profiles are shown in Fig. 2. No significant association was found among the proportion of PD-1, CCR7, or CD45RA expression in peripheral blood CD4 + T cells and survival (Table 2 and Table 3). Then, a subset analysis was performed according to the tumor PD-L1 expression level. Only the proportion of CD45RA-CD4 + T cells was associated with PFS in each group classified according to tumor PD-L1 expression level. Patients with a higher proportion of CD45RA-CD4 + T cells (≥ 30%) showed longer PFS times in the subsets with tumor PD-L1 TPS of ≥ 50% (median, not reached vs. 15.8 months, *p* = 0.167, log-rank test) and < 50% (median, 6.2 months vs. 1.5 months, *p* = 0.001, log-rank test), respectively (Fig. 3). Therefore, we analyzed the association of the proportion of CD45RA-CD4 + T cells with survival by multivariate analysis using the Cox proportional hazard model. Cox proportional hazard model for PFS adjusting for PS, PD-L1 TPS, EGFR mutation status and combined therapy with cytotoxic agents showed that the proportion of CD45RA-CD4 + T cells was significantly associated with a reduced risk of progression (Table 4, hazard ratio: 0.18, 95% CI: 0.04–0.78).

**Table 2:** Univariate analysis using log-rank test for progression-free survival.

		Median (months)	95% CI	*p*
CD45RA+CCR7+	< 50%	13.7	3.0–NE	0.092
	≥ 50%	4.4	1.5–12.1	
CD45RA+CCR7−	< 7%	6.8	1.5–12.1	0.624
	≥ 7%	6.3	1.7–19.5	
CD45RA−CCR7−	< 5%	6.3	1.5–12.1	0.328
	≥ 5%	12.2	1.7–NE	
CD45RA−CCR7+	< 25%	2.3	1.5–12.1	0.186
	≥ 25%	6.8	3.0–NE	
CD45RA−	< 30%	2.3	1.2–12.1	0.166
	≥ 30%	6.8	2.4–NE	
CCR7−	< 15%	6.8	1.7–NE	0.739
	≥ 15%	6.2	1.5–19.5	
PD-1+	< 30%	2.4	1.5–NE	0.514
	≥ 30%	8.9	3.0–19.5	

CI, confidence interval; NE, not estimated; PD-1, programmed death-1. Patients were subdivided according to the median of the proportion of each subset against CD3 + CD4 + T cells.

**Table 3:** Univariate analysis using log-rank test for overall survival.

	%	Median (months)	95% CI	*p*
CD45RA+CCR7+	< 50	NR	7.7–NE	0.220
	≥ 50	18.6	4.1–NE	
CD45RA+CCR7−	< 7	18.6	3.9–NE	0.509
	≥ 7	19.0	7.1–NE	
CD45RA−CCR7−	< 5	18.6	7.1–NE	0.816
	≥ 5	19.0	4.4–NE	
CD45RA−CCR7+	< 25	18.6	3.9–NE	0.479
	≥ 25	NR	7.1–NE	
CD45RA−	< 30	18.6	3.7–NE	0.461
	≥ 30	19.0	7.1–NE	
CCR7−	< 15	18.6	7.1–NE	0.826
	≥ 15	19.0	4.4–NE	
PD-1+	< 30	18.6	4.1–NE	0.474
	≥ 30	NR	7.7–NE	

CI, confidence interval; NE, not estimated; NR, not reached; PD-1, programmed death-1. Patients were subdivided according to the median of the proportion of each subset against CD3 + CD4 + T cells.

**Table 4:** Multivariate analysis using Cox proportional hazard model for progression-free survival.

		HR	95% CI	*p*
PS	0–1	1.66	0.50–5.45	0.407
	2	1.00		
PD-L1 TPS	≥ 50%	0.04	0.01–0.25	0.001
	Unknown	0.66	0.06–7.43	0.737
	< 50%	1.00		
EGFR	Positive	3.13	0.75–13.13	0.119
	Negative/unknown	1.00		
Combined therapy with cytotoxic agents	Yes	0.24	0.07–0.87	0.030
	No	1.00		
CD45RA-	≥ 30%	0.18	0.04–0.78	0.022
	< 30%	1.00		

CI, confidence interval; EGFR, epidermal growth factor receptor; HR, hazard ratio; PD-L1 TPS, programmed death ligand-1 tumor proportion score; PS, performance status.

**Figure 4:**
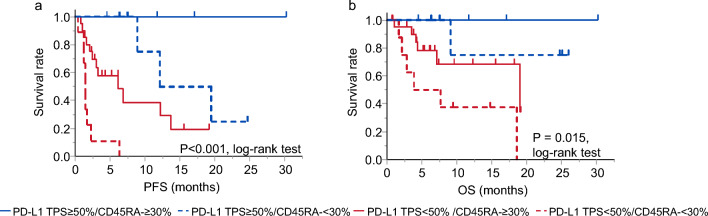
Progression-free survival (**a**) and overall survival (**b**) after the initiation of immune checkpoint inhibitor therapy in patient groups classified according to tumor PD-L1 expression level and proportion of peripheral CD45RA-CD4 + T cells. PD-L1 TPS, programmed death-ligand 1 tumor proportion score.

Figure 4 shows Kaplan–Meier curves for PFS and OS after the initiation of ICI therapy in patient groups classified according to the tumor PD-L1 expression level and proportion of the CD45RA-CD4 + T cells. Those with a higher proportion of CD45RA-CD4 + T cells (≥ 30%) showed longer PFS and OS times than those with a lower proportion, in each group classified according to tumor PD-L1 expression level.

### Association with clinical parameters

Table 5 shows a comparison of patient characteristics and the proportion of CD45RA-CD4 + T cells. The proportion of CD45RA-CD4 + T cells was significantly higher (≥ 30%) in the patients who received ICI therapy as a first-line treatment.

**Table 5:** Comparison of patient characteristics between those with higher and lower proportions of peripheral CD45RA-CD4 + T lymphocytes.

	CD45RA ≥ 30%		*p*
	N	%	
Age (yr)			
< 75	11/18	61.1	1.000
≥ 75	14/22	63.6	
Sex			
Male	18/31	58.1	0.440
Female	7/9	77.8	
Smoking history			
Yes	22/34	64.7	0.654
No	3/6	50.0	
Histology			
Adeno	18/28	64.3	0.736
Squamous	7/12	58.3	
Driver mutation			
EGFR	2/6	33.3	0.285
KRAS	4/5	80.0	
Negative/unknown	19/29	65.5	
PD-L1 TPS			
< 50%	21/29	72.4	0.082
≥ 50%	4/10	40.0	
PS			
0–1	19/33	57.6	0.224
2	6/7	85.7	
Stage			
IVA or earlier	18/26	69.2	0.310
IVB	7/14	50.0	
NLR			
< 5	15/24	62.5	1.000
≥ 5	10/16	62.5	
Treatment line of ICI			
1	24/34	70.6	0.021
≥ 2	1/6	16.7	

EGFR, epidermal growth factor receptor; ICI, immune checkpoint inhibitor; NLR, neutrophil–lymphocyte ratio; PD-L1 TPS, programmed death ligand-1 tumor proportion score; PS, performance status.

## Discussion

The present study showed that the proportion of CD45RA-CD4 + T cells was associated with PFS after the start of ICI therapy, independent of several clinical background factors, indicating that peripheral CD4 memory T cells are associated with the efficacy of ICIs for treating NSCLC patients.

In the tumor microenvironment, CD8 + T cells are associated with the elimination of cancer cells, and it has been reported that tumor-infiltrating CD8 + T cells are associated with the efficacy of ICI therapy^18,19^. On the other hand, from the studies investigating peripheral immune cells, several authors have reported the association between CD4 + T cells and the efficacy of ICI therapy^8–11^, while the association with CD8 + T cells was also reported^20,21^. Furthermore, it was recently reported that CD4 + T cells in the tumor microenvironment are also associated with longer survival after the initiation of ICIs^22^. Given that CD4 + T cells play an important role in the differentiation and activation of CD8 + T cells under the support of dendritic cells^23^, they might be involved in the efficacy of ICIs.

The findings of the present study suggested the contribution of memory CD4 + T cells to the efficacy of ICI therapy. Similar results have been previously reported. Kagamu H et al. reported that CD62L^low^ T cells were related to the response to ICI therapy. Low expression of CD62L, a homing receptor, indicates the presence of antigen-primed T cells. The percentage of CD62L^low^CD4 + T cells was also significantly correlated with the percentage of effector memory T lymphocytes that are identified as CCR7-CD45RA-^8^. Zuazo M et al.^9^ reported that highly differentiated CD4 + T cells, defined as CD27-CD28- in peripheral blood, were associated with PFS after the initiation of ICIs and that the majority of these T cells were central memory (CD45RA-CD62L +) or effector memory T cells (CD45RA-CD62L-). Iwahori et al. reported that peripheral blood T-cell cytotoxicity is an independent prognostic factor for PFS and that peripheral T-cell toxicity is correlated with the ratio of the effector memory population in CD4 + or CD8 + T cells^24^. Miao et al.^10^ reported that CD45RA-CD4 + T cells were associated with PFS in patients with NSCLC who received ICI therapy. Notably, memory T cells are thought to be necessary for long-term immunological memory and possibly for lasting response^25^. These reports on the association between CD4 memory T cells and the efficacy of ICI therapy suggest that the existence of antigen-primed CD4 T cells is related to the efficacy of ICIs. However, Zuazo M reported that the proportions of CD4 T cells that were reactive to A549 cell antigens were not significantly different between differentiated and non-differentiated T-cell groups^9^.

We previously reported that the proportion of PD-1 + CD4 + T cells evaluated within 1 week after initiation of ICIs was associated with the efficacy of ICIs. These findings were similar to those reported by Waki K et al.^15^ and Santegoets et al.^26^; however, no such relationship was observed in the present study. In the previous study, we obtained peripheral blood after ICI administration in 17 (89.5%) patients, but herein, it was obtained before the treatment in 37 (92.5%) patients. Du W et al. reported that responders to ICIs showed an increase in PD-1 + CD4 + T cells after one cycle of ICI therapy^27^. These findings suggest that an increase of PD-1 + CD4 + T cells after the initiation of ICI administration, but not at baseline, might be associated with the response to ICI therapy.

There are several limitations in the present study, and the findings should be interpreted with caution. First, the sample size was small, which may raise the issue of generalizability. Second, even if the peripheral memory CD4 + or differentiated CD4 + T cells are important for the efficacy of ICIs, it remains unclear whether CD45RA is the optimal marker for its evaluation. Furthermore, the optimal cutoff value for CD45RA-CD4 + T cells cannot be determined in the present study. Finally, although some clinical factors were adjusted by multivariate analysis, the patient backgrounds and treatments were not well balanced among the groups.

In summary, the present study suggested that peripheral CD4 + memory T cells, defined as CD45RA-CD4 + , are independently associated with PFS after the initiation of treatment with ICIs.

## Data Availability

The datasets analyzed during the current study are available from the corresponding author on reasonable request.
